# Accounting for Redundancy when Integrating Gene Interaction Databases

**DOI:** 10.1371/journal.pone.0007492

**Published:** 2009-10-22

**Authors:** Antigoni Elefsinioti, Marit Ackermann, Andreas Beyer

**Affiliations:** 1 Biotechnology Center, TU Dresden, Dresden, Germany; 2 Center for Regenerative Therapies Dresden, TU Dresden, Dresden, Germany; University of Glasgow, United Kingdom

## Abstract

During the last years gene interaction networks are increasingly being used for the assessment and interpretation of biological measurements. Knowledge of the interaction partners of an unknown protein allows scientists to understand the complex relationships between genetic products, helps to reveal unknown biological functions and pathways, and get a more detailed picture of an organism's complexity. Being able to measure all protein interactions under all relevant conditions is virtually impossible. Hence, computational methods integrating different datasets for predicting gene interactions are needed. However, when integrating different sources one has to account for the fact that some parts of the information may be redundant, which may lead to an overestimation of the true likelihood of an interaction. Our method integrates information derived from three different databases (Bioverse, HiMAP and STRING) for predicting human gene interactions. A Bayesian approach was implemented in order to integrate the different data sources on a common quantitative scale. An important assumption of the Bayesian integration is independence of the input data (features). Our study shows that the conditional dependency cannot be ignored when combining gene interaction databases that rely on partially overlapping input data. In addition, we show how the correlation structure between the databases can be detected and we propose a linear model to correct for this bias. Benchmarking the results against two independent reference data sets shows that the integrated model outperforms the individual datasets. Our method provides an intuitive strategy for weighting the different features while accounting for their conditional dependencies.

## Introduction

One of the basic aims of the post-genomic era is the construction of reliable and accurate interactomes for different organisms and especially for human. Such interaction maps allow for understanding the complex relationships between genetic products, help to reveal unknown biological functions and pathways, and get a more detailed picture of an organism's complexity [1], [2].

High throughput techniques, such as yeast two-hybrid (Y2H) or mass spectrometry-based proteomics (AP-MS), led to the construction of large protein-protein interaction (PPI) networks. For example in case of the human interactome, approximately 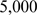 interactions were characterized by using Y2H [3], [4] and close to 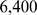 by using AP-MS techniques [5]. Together with the PPIs that have been characterized in small-scale experiments [6], the sum of the experimentally determined physical interactions does not exceed 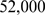. This is less than 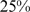 of the estimated human interactome that is predicted to include between 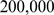
–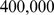 interactions [7], [8].

In order to deal with this lack of information different methods for predicting interactions have been developed leading to the creation of databases, which integrate known and predicted interactions from various data sets. Such data integration improves the coverage and the specificity of interaction predictions [2], [9]. Different evidences may cover different interaction types and interactions supported by diverse evidences are of higher confidence. Furthermore, it has been shown that integrating data from a variety of sources allows for reliably predicting interactions even though individual evidences may only be weak predictors if taken alone [10], [11].

Because of the exceptional importance of human interaction data in the life sciences we sought for combining predictions of human gene interactions from different sources. Such ‘meta interactome’ should combine the information retrieved from various interaction databases in a consistent way and thus establish a more comprehensive map of the human interactome.

**Figure 1:**
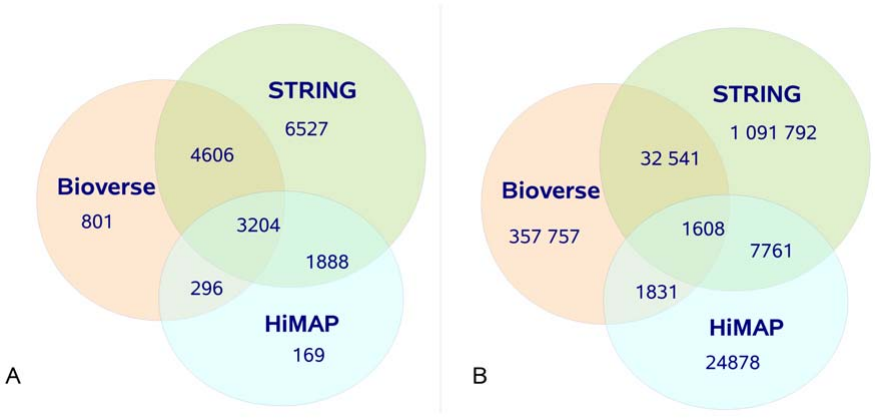
Venn diagram of the overlap between Bioverse, HiMAP and STRING. (A) Overlap between genes. Approximately 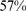 of the genes are common to at least two databases. (B) Overlap between interactions. Only 2.8% of the interactions are reported in more than one database.

STRING [12], HiMAP [13] and Bioverse [14] are examples of databases integrating evidence for gene interactions in human and other species. Those databases report quantitative confidence scores for each interaction, with higher scores reflecting higher likelihood that the given interaction is real. The total number of genes and interactions in each of the three databases is shown in Table 1. These databases use different prediction methods, experimental sources and different scoring schemes for quantitatively integrating this information. Even though all of these databases use partly overlapping input data, only 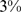 of the interactions are reported in more than one database (Figure 1). Hence, by combining the data from all three databases the size of the predicted human interactome could be significantly increased. In addition, the integration renders the overlapping interactions particularly confident. The size of this ‘core interactome’ (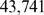) is similar to the total number of experimentally determined interactions. Here we present an integrated human interactome consisting of the combined data from all three databases. Since all three input databases predict functional association between genes also our integrated network reflects functional similarity of genes rather than physical binding of protein products. For integrating the scores from the different databases we employed a Bayesian approach that quantifies the likelihood of an interaction given all available evidences [10]. The main advantages of this method are that (i) it allows for combining highly heterogeneous types of data, such as categorical and numerical features, (ii) it weighs each information source according to its reliability, (iii) it provides a uniform scoring scheme for comparing and integrating databases, and (iv) scores have an intuitive meaning as they reflect conditional probability relationships between evidences and gold-standard data sets.

**Table 1:** Number of proteins and interactions in different databases.

	Proteins	Interactions
Bioverse	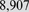	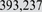
STRING	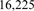	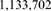
HiMAP	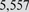	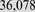

**Figure 2:**
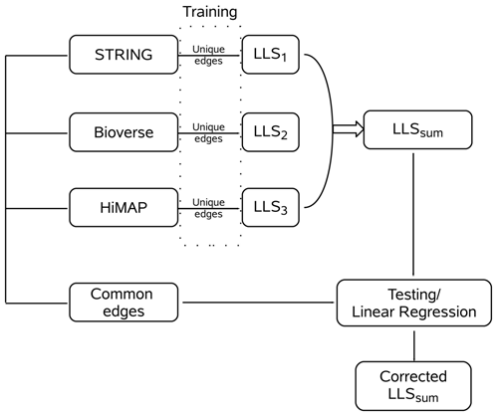
Diagram depicting the steps of log-likelihood score (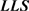) calculation. Initially log-likelihood scores were calculated for each database independently. A naive Bayes classifier was applied to the individual data sets for mapping the interaction confidence onto a common scale. Subsequently a linear correction was applied to 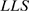 obtained from more than one database.

An important assumption of the ‘Naive Bayesian Integration’ is, however, that all evidences used are statistically independent [15]. Previous work has shown that dependencies between the input data exist, but that such correlations do not pose a major confounding effect [10]. This previous work was assessing the dependence between different types of evidences, such as independent measurements of the same protein interaction. However, here we are integrating data from different databases that partially rely on identical data (e.g. gene expression measurements or text mining). Hence, the assumption of independence may strongly bias the interaction predictions in our case. We therefore developed a method for correcting for the bias introduced when combining statistically dependent data with a Bayesian approach in order to create a continuous scale confidence score that is comparable between interactions with common and unique evidences (Figure 2).

## Results

### 0.1 Cross Validation

**Figure 3:**
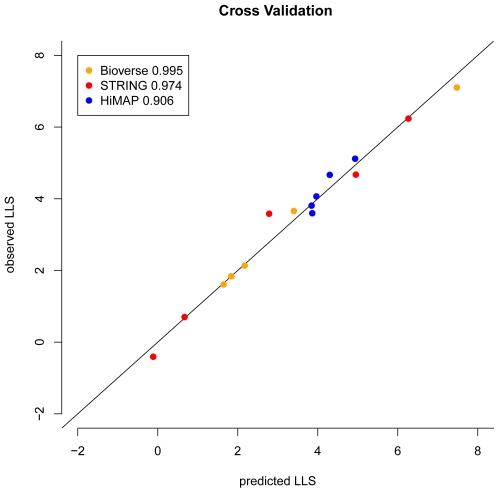
Cross validation. Three-fold cross validation was applied for each of the three databases independently. Training of the 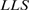 prediction was done based on two-thirds of the reference data. The x-axis represents the predicted 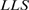 from the training parameters while the y-coordinate represents the actual enrichment with true positives in the test set. The data were binned in five bins and the dots show the respective 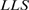 for each bin. The color indicates the source database. For all three datasets predicted and observed results are very close to the ideal case (solid line). The correlation coefficients between predicted and true 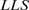 are reported in the figure legend.

We applied Bayesian theory to the original scores provided by Bioverse, HiMAP and STRING in order to calculate log-likelihood scores (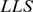) for each of these three data sets independently. This model assumes independence between different evidences, in order to train the input data for predicting PPIs. A three-fold cross validation was implemented for evaluating the Bayesian model. The cross validation was done by training the 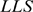 computation based on two thirds of the reference dataset. Subsequently, 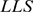 were computed for the remaining interactions and compared to the actual enrichment of true positives in the test set (Figure 3). If the 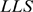 prediction works perfectly, we expect a 1∶1 relationship between the predicted 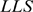 and the actual enrichment of true positives. Figure 3 shows that, when assessing individual databases, the predicted 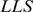 coincide with the actual enrichment of true positives in the test set, thereby validating the accuracy of the method.

### 0.2 Integrating interactions with more than one evidence

When combining scores for interactions that are reported in more than one database we have to consider the issue of statistical dependence between the evidences. The so-called Naive Bayesian Integration relies on the assumption of independence by simply multiplying the probability ratios (see Methods):
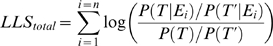
(1)


However, for correlated evidences this assumption would yield an overestimation of the true interaction likelihood.

In order to assess the correlation structure between the databases we divided the interactions that are reported in at least two databases into four different subgroups: (i) interactions that are reported in Bioverse and STRING but not in HiMAP, (ii) interactions that are reported in Bioverse and HiMAP but not in STRING, (iii) interactions that are reported in STRING and HiMAP but not in Bioverse and, finally, (iv) interactions that are reported in all three databases.

**Figure 4:**
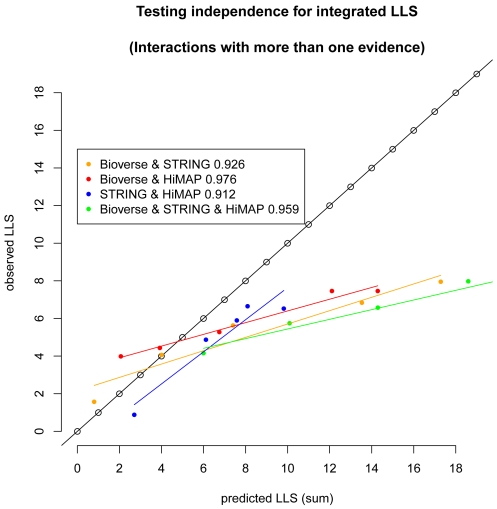
Estimating feature dependencies. The plot of observed versus estimated 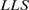 for all four subgroups of integrated interactions illustrates the dependencies between the three different datasets as well as the linear correlation between predicted and observed log-likelihood scores. The approach is the same in as Figure 3 with the difference that the test set is limited to common interactions. Linear correlation coefficients (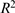) are reported in the legend.

For each of these subsets we computed integrated 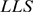 by initially assuming independence, i.e. we applied equation 1. In order to avoid circular reasoning we trained the individual 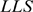 for each database on interactions that are present in only one database, predicted the 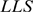 of interactions that are common (i.e. from the sets (i) – (iv) above) and computed the integrated predicted 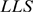 by applying equation 1 (Figure 4). However, because the fraction of common interactions is quite small, the training is almost identical when including interactions that are common to more than one database (not shown).

If the information in the three datasets was non-redundant (i.e. if the evidences were truly independent) the predicted scores would perfectly match the observed ones, like in the cross validation shown above (Figure 3). However, Figure 4 clearly shows a systematic bias. The regression lines show a systematically increasing overprediction of the true likelihood scores.

If two databases use the same data source for predicting an interaction, the naive Bayes model accounts twice for the same information, which leads to an overestimation of the true interaction probability. Figure 4 also shows that the bias is very similar for the different combinations of interactions reported in two databases (i–iii), suggesting that the degree of redundancy is similar among them. Consistently, interactions that are reported in three databases tend to be overestimated even more than interactions reported in two databases.

For further investigating the sources of redundancy we calculated the correlations of scores from the input databases for the portion of the redundant data (Table 2). Unfortunately, STRING is the only database that provides details on how individual interaction scores are composed. Bioverse and HiMAP provide only an integrated score, making it impossible to assess the correlation between individual evidences in the different data sources, or to eliminate the common ones. The correlation coefficients show no correlation between Bioverse an HiMAP and the different STRING inputs, suggesting that we cannot just exclude inputs from the redundant data sources in order to correct for the bias.

**Table 2:** Correlation of scores from different evidences.

	neigh	fus	cooc	coexp	exper	dbase	txt	STRING	HiMap	Bioverse
neigh	-	0.3256	0.2118	0.1068	0.1191	0.0137	0.0383	0.1869	0.018	0.0052
fus	-	-	0.0816	0.0247	0.0057	6e-04	0.0039	0.1	0.0042	4e-04
cooc	-	-	-	0.0156	0.0186	0	0.0814	0.1407	2e-04	0.0036
coexp	-	-	-	-	0.0243	0.0101	0.0027	0.0015	0.005	0.006
exper	-	-	-	-	-	0.0082	0.1384	0.5073	0.0264	0.1158
dbase	-	-	-	-	-	-	0.0045	0.2386	0.0057	2e-04
txt	-	-	-	-	-	-	-	0.6146	0.0313	0.1043
STRING	-	-	-	-	-	-	-	-	0.028	0.1946
HiMap	-	-	-	-	-	-	-	-	-	0.0199

The linear correlation coefficients (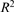) for the redundant interactions. Information for individual experimental inputs is available only for STRING. The first eight columns correspond to the individual STRING datasources, while the column named ‘STRING’ refers to the integrated score provided by the database. Bioverse and HiMAP provide only one integrated score. Abbreviations. neigh: neighborhood, fus: fusion, cooc: cooccurence, coexp: coexpression, exper: experimental, dbase: database, txt: textmining.

Though the observed scores for integrated evidence do not coincide with the predicted ones (as shown in Figure 4), the bias is linear in the log-space. This observation suggests applying a linear correction of 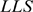 that are based on more than one database in order to account for the redundancy between the databases.

Thus, after summing the 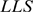 from the individual databases, we perform a linear regression against the actual enrichment of true positives as shown in Figure 4. The regression parameters (slope and intercept) are then used to correct the predicted 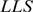. Importantly, we perform a separate regression and correction for each database pair and for interactions common to all three databases.

### 0.3 Assessing the linear bias correction

**Figure 5:**
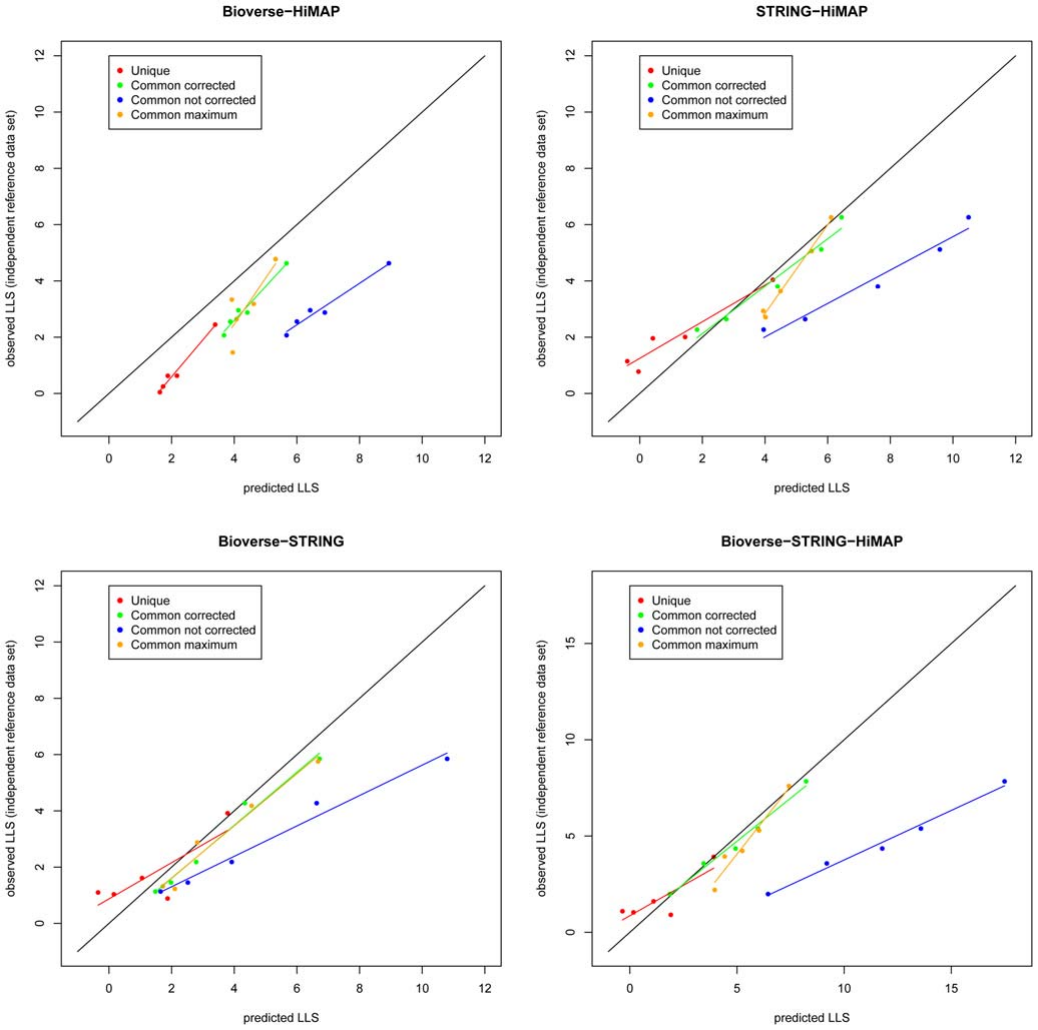
Assessing the linear bias correction. Linear regression plots for trained (predicted) versus tested 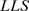 based on a second, independent reference data set for the different combinations of redundant subsets. Red line: interactions reported in only one database. Green and blue line: corrected and uncorrected 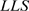 for interactions reported in at least two databases. Orange line: Using the maximum of the individual 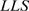 instead of the sum. Ideally, all predictions should be along the diagonal. The bias corrected scores are clearly better predictors of the true interaction likelihood.

In order to independently quantify the impact of the dependency between the data sources on our prediction and for scoring the success of our correction, we could not apply a cross validation, due to the small overlap between the common interactions and the ‘Gold Standard’. Thus, we utilized three other sets of interactions that are based on experimental evidence but are distinct from our previous reference dataset. The first one consists of human interactions from the MIPS CORUM database (Comprehensive Resource of Mammalian protein complexes) [16]. The MIPS CORUM database is a resource of manually annotated protein complexes from mammalian organisms. We used the Core Set, that is a reduced dataset which is essentially free of redundant entries, and consists of 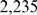 proteins. After using the ‘matrix model’ we obtained 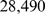 binary interactions. The ‘matrix model’ [17] assumes that any two proteins within a complex have a pairwise interaction. The second dataset is derived from HPRD and includes protein interactions measured *in vitro* or with Y2H experiments, after excluding all interactions that were already used in the previous positive reference set. The last dataset is derived from IntAct [18] by using interactions that are described as ‘Physical interactions’ in the field ‘Interaction type’. We merged these three datasets and obtained a total of 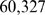 interactions. In order to test the success of our correction for the four different redundant subsets we split this second benchmark dataset in two subsets. The first one contains all interactions reported in only one of the input data sets (‘unique interactions’) and the second one consists of interactions reported in more than one database (‘common interactions’). Ideally, the predicted 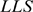 should be close to the true one and the predictive performance of the training based on HPRD *in vivo* interactions should be the same for both, common and unique interactions. A large difference between ‘unique’ and ‘common’ indicates a bias. If the data were completely correlated, using the maximum instead of the sum of the 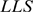 would be a better predictor of the true likelihood. In order to asses the correlation between different common evidences and compare the ‘maximum score’ approach with our corrected bias method we tested the success of this approach for the ‘common interactions’ dataset. Figure 5 shows the correlation between the 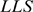 obtained after training with the HPRD *in vivo* interactions and the actual enrichment of true positives using our second benchmark data set. The corrected 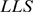 of the common interactions are more consistent with scores from the unique interactions and they are closer to the unity line (diagonal) than the uncorrected sum of scores (Naive Bayesian approach) or the maximum. Hence, the linear correction is successfully reducing the bias introduced by the partial redundancy between the databases. Using the maximum also reduces the overprediction bias considerably. However, in several cases the scores are less consistent with the scores of the ‘unique’ edges (Figure 5). This is an issue when scores between ‘unique’ and ‘common’ edges are compared in subsequent analyses. This assessment is also supported by the findings from the correlation between different individual data sources (Table 2). The ‘maximum approach’ assumes a perfect correlation between the features, which is not the case for our data. Finally this assessment shows that the correction is not strictly dependent on a single training dataset.

### 0.4 Evaluating the predictive power of the bias correction

**Figure 6:**
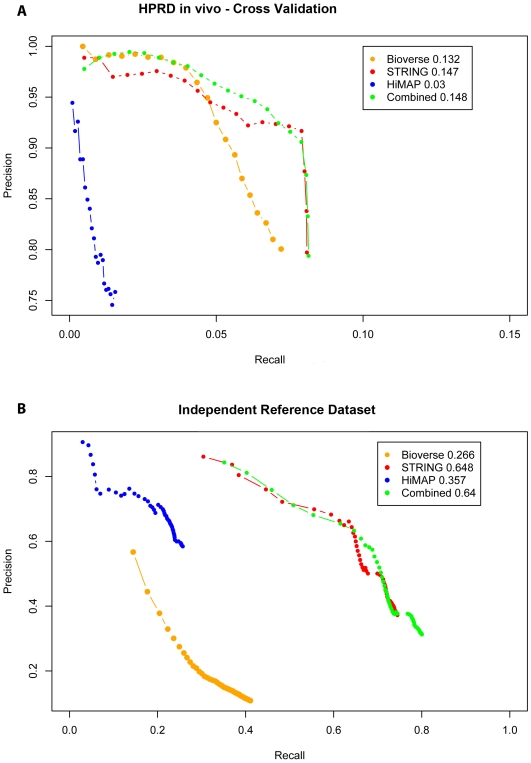
Benchmarking the integrated interactome. (A) Cross validation based on the HPRD *in vivo* reference set. (B) Training on HPRD *in vivo*, testing on independent reference set (see main text for details). We divided the test dataset in 20 bins based on their descending log-likelihood score and assessed the cumulative precision and recall for each successive bin for the corrected score and for the scores derived from training the individual databases. The integrated network shows equal or better overall performance. The maximum F-score of each network is reported in the legend. The F-score (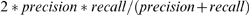) is an integrated measure of the predictive power.

After training the Bayesian predictor and applying the bias correction we set out to assess the predictive power of this method for the interactions having at least two evidences. We applied a three-fold cross validation to assess our bias correction. The training sets were used for computing the correction parameters and the success of the correction was subsequently tested on the remaining edges. Additionally, we tested the integrated interactome, along with the three individual interactomes, on the second, independent reference interactions from above (HPRD non*-in vivo* interactions, MIPS CORUM, and IntAct). Figure 6 shows the cumulative precision and recall of the three individual databases and the integrated scoring after correcting for the dependency between the databases for both cases.

In order to assess the generality of the bias correction across the genome and to avoid pair associated biases we measured the recall of genes rather than interactions [11]. The integrated model clearly outperforms any of the individual databases in both cases. In particular it achieves a better precision than any of the individual databases at almost any level of recall. When testing on the independent reference set, the integrated interactome achieves a recall of 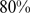, which is significantly higher than the maximum recall of any of the individual databases (STRING: 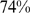, Bioverse: 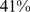, HiMAP: 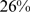).

## Discussion

By combining the information from different databases that independently predict gene interactions it is possible to significantly increase the coverage and significance of such networks. Even though such databases rely on partially overlapping information they score largely distinct sets of interactions.

In this study, we integrated information from three different interaction databases, STRING, Bioverse and HiMAP and created a joint network consisting of 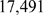 human genes and 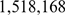 interactions. In order to map the confidence scores from the individual databases onto a common probability scale we used a Bayesian approach. A Bayes classifier has the benefit of easily integrating diverse features, dealing with missing values and being readily interpretable and computationally inexpensive.

One of the major considerations when combining different features was whether or not we should take conditional dependencies into account. Lu et al. [19] suggest that correcting for conditional dependencies, when predicting protein interactions, does not significantly improve the performance. Specifically they addressed this question by comparing the performance of a simple Naive Bayes classifier (SNB) with that of a boosted Naive Bayes classifier (BNB) [20]. Boosting approximates the best linear combination of all possible weak classifiers (e.g different features) via maximum likelihood on a logistic scale, thereby solving statistical dependence problems [21]. Thus, a boosted Naive classifier is more resistant to redundant information between evidences. However, Lu et al. showed that their SNB performs almost as well as the BNB for weakly dependent yeast protein-protein interaction data. Hence, previous work was suggesting that the dependence between evidences can largely be ignored when using a Bayesian classifier for predicting gene interactions [22]. The main difference between previous work and this study is that we are integrating databases that are partly relying on identical data. Our study shows that the conditional dependency cannot be ignored when combining databases that rely on partly overlapping input data. Fortunately, this bias can be removed by applying a simple linear correction to the integrated log likelihood scores derived from the naive Bayes classifier. When comparing the prediction accuracy of the Naive Bayesian approach against the corrected Bayesian approach, the latter was performing significantly better. This is important because, though the interactions that are reported in more than one database cover only 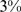 of the total predicted interactome, they are creating a network of 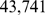 high scoring interactions which is important for follow up analyses requiring high confidence interactions. Further, only after applying our correction, scores of ‘common’ interactions become comparable to scores of ‘unique’ interactions.

## Materials and Methods

### 0.5 Data Collection

Information about gene associations was collected from STRING [12], [23] (version 8.0), HiMAP [13] (version 1.0), and Bioverse [14], [24] (version 2.0). No filtering criteria were applied when integrating the three databases, i.e. even low-scoring interactions were included. All gene identifiers were mapped to ENSEMBL gene IDs. Interactions involving genes that have no ENSEMBL gene ID were discarded.

**STRING** uses a variety of methods for predicting protein associations: Genomic Context (like gene fusion, phylogenetic co-occurrence, conserved neighborhood), high-throughput experiments (Y2H), conserved co-expression in different conditions, previous knowledge (database imports and and literature co-occurrence), and observed interaction of orthologous genes in other species. A final combined (Bayesian) score is calculated to integrate the individual scores.

**HiMAP** includes approximately 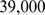 interactions of human genes, which are predicted based on model organism interactions (*Saccharomyces cerevisiae* [25]–[28], *Drosophila melanogaster* [29] and *Caenorhabditis elegans* [30]), co-expression matrices from ONCOMINE [31], [32], shared biological function from Gene Ontology [33] and information about enriched domain pairs from InterPro [34].

**Bioverse** uses a method for predicting protein-protein interactions similar to the interolog method [35]–[37]. Interactions were predicted when each member of an experimentally derived interaction was found to be similar with different interaction candidates in the query interactome. A score based on the similarity score was calculated for assessing the interaction probability.

### 0.6 Reference dataset

Like any other supervised method, Bayesian integration requires a reference (‘Gold Standard’) data set (both for positive and negative interactions). Such set of trusted reference interactions should have a sufficient size which allows for statistically reliable predictions, no systematic bias, and be as reliable as possible. We used interactions from the Human Protein Reference Database (HPRD) reported as *in vivo* as our gold-standard for positive interactions (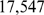 interactions in total). By using *in vivo* interactions only we constrained the positives to a set of well defined and accurate PPIs.

The construction of negative training sets for protein interaction prediction is a notorious problem [10], [38], because many interactions that are not reported in the databases may actually be true positives due to our incomplete knowledge. We therefore restricted the construction of the negative training set to the same proteins as in the positive training set. Given that the proteins in the HPRD *in vivo* dataset have been individually studied we reasoned that comparably few interactions of these proteins are unknown. Hence, interactions among these proteins that are not in our positive control set, are more likely to be true negative interactions.

### 0.7 Log-likelihood calculation - Bayesian approach

In order to combine information from the three different data sets we calculated log-likelihood scores as described previously [10], [22]. Likelihood scores quantify the ability of evidences to predict protein interactions by measuring the ratio of ‘true to false number of interactions’ for a benchmark set with that specific evidence.

Each of the three input databases is considered as distinct evidence and initially the predictive power of all three evidences is estimated independently from each other. According to the Bayesian rule the posterior odds of interaction are analogous to the prior odds and the likelihood score (ratio, 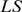)
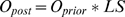
(2)where
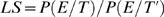
(3)

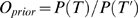
(4)and
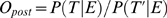
(5)


Hence, when transforming to logarithmic scale we get:
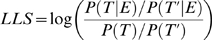
(6)where 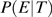 and 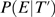 are the probabilities of an interaction to have the evidence, given that the interaction is true or false (to belong to the positive or the negative reference data set) and 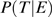 and 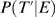 are the probabilities of an interaction to be true or false, given the evidence exists, while 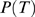 and 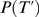 represent the prior probabilities of an interaction to belong to the positive or negative set, respectively, in comparison to the whole interaction space. The prior probabilities are obtained from the fractions of positive and negative interactions in the reference set. Note that equation 2 computes the ratio of two Bayes factors. Log-likelihood scores can be used as uniform scoring schemes between interactions and as measures for weighting each individual feature according to its reliability. 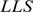 with values greater than zero indicates that interactions with the given evidence score are more likely to be true than false interactions.

Calculating 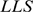 means adjusting different evidences to a common benchmark. That makes the different scores comparable even if they initially are of a different nature.

In order to train for log-likelihood calculation we used the interactions that are present both in the dataset serving as evidence and in the positive or negative reference datasets. These interactions were binned, while ensuring that each bin includes the same number of interactions. Binning allows us to estimate the conditional expectation of the dependent variable given the independent variable, i.e. apply equation 6. After calculating the 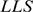 for each bin we applied a linear regression using the original database scores as the independent and the 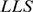 trained on our common reference set as the dependent variable. This regression was subsequently used for predicting the 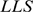 of new interactions (i.e. interactions not part of the reference dataset).

### 0.8 Linear bias correction

We applied a linear regression between observed and predicted scores for interactions with more than one evidence in order to correct for feature dependencies. First, we computed a predicted 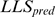 by summing the 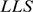 from the individual databases (Naive Bayesian model),
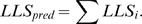
(7)


Next, we computed the correlation between the predicted and true 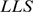 based on the ‘common’ interactions,
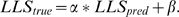
(8)


Finally, the parameters 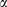 and 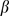 were used for correcting for the bias.
